# Postpartum Interventions to Reduce Long-Term Cardiovascular Disease Risk in Women After Hypertensive Disorders of Pregnancy: A Systematic Review

**DOI:** 10.3389/fcvm.2019.00160

**Published:** 2019-11-15

**Authors:** Nicla A. Lui, Gajana Jeyaram, Amanda Henry

**Affiliations:** ^1^Department of Women's and Children's Health, St. George Hospital, Sydney, NSW, Australia; ^2^School of Women's and Children's Health, University of New South Wales, Sydney, NSW, Australia; ^3^The George Institute for Global Health, Sydney, NSW, Australia

**Keywords:** cardiovascular risk reduction, pre-eclampsia, gestational hypertension, hypertensive disorders of pregnancy, systematic review, cardiovascular disease, lifestyle behavior change

## Abstract

**Introduction:** Hypertensive disorders (HDP) affect ~7% of pregnancies. Epidemiological evidence strongly suggests HDP independently increases that individual's risk of later cardiovascular disease (CVD). Focus on reduction or mitigation of this risk has been limited. This review seeks to identify trialed interventions to reduce cardiovascular risk after HDP.

**Methods:** Online medical databases were searched to identify full-text published results of randomized controlled trials (RCT) in women <10 years postpartum after HDP that trialed interventions to reduce cardiovascular risk. Outcomes sought included cardiovascular disease events, chronic hypertension, and other measures of cardiovascular risk such as obesity, smoking status, diet, and physical activity. Publications from January 2008 to July 2019 were included.

**Results:** Two RCTs were identified. One, a trial of calcium vs. placebo in 201 women with calcium commenced from the first follow-up visit outside of pregnancy and continued until 20 weeks' gestation if another pregnancy occurred. A non-significant trend toward decreased blood pressure was noted. The second RCT of 151 women tested an online education programme (vs. general information to control group) to increase awareness of risk factors and personalized phone-based lifestyle coaching in women who had a preeclampsia affected pregnancy in the 5 years preceding enrolment. Significant findings included increase in knowledge of CVD risk factors, reported healthy eating and decreased physical inactivity, however adoption of a promoted heart healthy diet and physical activity levels did not differ significantly between groups. Several observational studies after HDP, and one meta-analysis of studies of lifestyle interventions not performed specifically after HDP but used to extrapolate likely benefits of lifestyle interventions, were identified which supported the use of lifestyle interventions. Several ongoing RCTs were also noted.

**Discussion:** There is a paucity of intervention trials in the early years after HDP to guide evidence-based cardiovascular risk reduction in affected women. Limited evidence suggests lifestyle intervention may be effective, however degree of any risk reduction remains uncertain.

**Conclusion:** Sufficiently powered randomized controlled trials of appropriate interventions (e.g., lifestyle behavior change, pharmacological) are required to assess the best method of reducing the risk of cardiovascular disease in this at-risk population of women.

## Introduction

Preeclampsia [PE] and hypertensive disorders of pregnancy [HDP] can now be acknowledged as independent risk factors for later cardiovascular disease (CVD) (1, 2). PE is a multisystem disorder diagnosed at >20 weeks' gestation with evidence of hypertension and involvement of one or more other organ systems (3). PE remains a leading cause of maternal death globally as well as serious morbidity for mother and baby (4).

Several systematic reviews of cohort and case control studies, including over 100,000 women post-PE and 2 million after normotensive pregnancy, have found that after PE, women have at least triple the risk of chronic hypertension, and double the risk of ischemic heart disease, stroke, and death related to cardiovascular disease, than their normotensive pregnancy counterparts (1, 5–7). Where confounding of other risk factors was able to be accounted for, this effect persists, strongly suggesting that history of PE is an independent risk factor for CVD. Although the absolute risk of cardiovascular disease in young women is small, the existing data would also suggest that the increased CVD relative risk is already present in the first 10 years postpartum (7) and continues lifelong (8). Although data is less extensive for gestational hypertension (GH), new-onset hypertension in the second half of pregnancy without the multisystem features of PE, it also appears to be associated with an approximately doubling of CVD risk (8, 9). Chronic hypertension in pregnancy (CH), hypertension already existing <20 weeks gestation, flags women already at increased risk of CVD. Given the prevalence of HDP in the population, affecting 7–10% of pregnancies (PE 2–5%, GH 4–6%, CH 1–2%) (4, 10), in combination with CVD being the largest cause globally of female mortality, the potential burden of disease is significant.

This phenomenon can be paralleled to the increased risk of Type 2 diabetes (T2DM) seen in women whose pregnancies have been affected by gestational diabetes (11). An increasing awareness of this risk has seen the encouragement to maintain lifestyle modifications that have been shown, albeit mostly in trials performed some years postpartum, to lower the risk of progression to T2DM, and the implementation of stricter follow-up policies to identify early development of changes to glucose metabolism (12, 13). A similar approach needs now to be considered to implement risk-reduction strategies and closer follow-up for women who have experienced a pregnancy affected by hypertensive diseases, in order to decrease CVD risk. Indeed, the latest International Society for the Study of Hypertension in Pregnancy (ISSHP) guidelines recommend follow-up of all women after HDP, assessment of CVD risk factors, education regarding long-term CVD risks associated with HDP, and counseling regarding lifestyle modification (3). However, the guidelines also acknowledge the paucity of the evidence base for what recommendations to give women and interventions to institute to lower CVD risk after HDP and note ongoing studies may give more guidance in this area.

The purpose of this systematic review is therefore to identify and assess trialed interventions to reduce long-term cardiovascular risk in women whose baseline risk has been increased given a pregnancy affected by hypertensive disease.

## Methods

A systematic review was conducted, reviewing any trialed interventions to reduce long-term CVD risk in women whose baseline risk has been increased due to a pregnancy affected by HDP, according to the PRISMA guidelines for systematic reviews (14). The protocol was prospectively registered in the PROSPERO database (CRD 4201920072).

The included population was women enrolled <10 years postpartum after a pregnancy affected by PE or GH, enrolled into any intervention trial to reduce cardiovascular risk. Women who were known to have another primary cause for hypertension or essential hypertension were excluded as these represent a group already known to be at increased CVD risk pre-pregnancy.

The search was limited to randomized controlled trials (including other trials where any randomization has occurred, including crossover and cluster), where an intervention aimed at reducing long term cardiovascular risk after GH or PE was interrogated. Postpartum intervention trials primarily based on another group, such as gestational diabetes or high BMI, which incidentally reported on women with previous HDP, were not included. There were no limitations on the nature of the intervention (e.g., life-style based, pharmacological or other). A control group not receiving the intervention was expected.

Primary outcomes were identified as a cardiovascular disease events or the development of chronic hypertension after the index pregnancy. Secondary outcomes, of (a) risk markers for CVD, and (b) markers of compliance (in order to assess feasibility of the trialed interventions) are listed in Table 1.

**Table 1:** Secondary outcomes considered.

**Secondary outcomes**	
Modifiable	Obesity (BMI ≥ 30 and/or waist circumference)
	Smoking status, however measured (e.g., self-reported, salivary cotinine)
	Diabetes/Impaired glucose tolerance
	Exercise participation, as measured by improvement in fitness scores using controlled fitness tests
	Diet quality, as recorded in food diaries
Compliance	Barriers to engaging in lifestyle modification programmes, as defined by patient questionnaire
	Compliance with long term medical therapy if prescribed, as measured by pill count and/or patient questionnaire.
	Compliance of patients with long term follow-up, as defined by attendance records

The systematic review was carried out by two independent reviewers who employed a pre-defined search strategy to interrogate online medical databases (MEDLINE, EMBASE, PubMed, Cochrane Database, Cochrane Register of Controlled Trials) to identify relevant trials (Appendix 1). There were no conflicts requiring the mediation of a third party. There were no language restrictions on initial results. Publications from January 2008 were included to ensure relevance (as the first meta-analysis of CVD risk after preeclampsia was published in 2007, it was not expected intervention trials meeting inclusion criteria would appear until after this time). On completion of the formal search strategy, titles and abstracts were reviewed to ensure inclusion criteria are met. Full text documents were obtained and assessed. Reference lists of included studies were searched to identify further studies, and a *post-hoc* decision was made to also search clinical trial registries (clinicaltrials.gov and all primary registries listed in the WHO International Clinical Trials Registry platform https://www.who.int/ictrp/network/primary/en/) for any further relevant studies.

A standardized form was created to compile a pre-specified set of extracted data of those eligible studies. Extracted data included: study setting, participant demographic details, details pertaining to the intervention and control, study methodology, recruitment and loss to follow-up rates, identification of barriers to completion of study, outcomes, and an assessment of risk of bias. Two reviewers independently assessed each study, with a plan to resolve discrepancies by way of discussion or deferring to a third reviewer, however this was not necessary. Any missing data would have been sought from study authors as required.

Each of the two individual reviewers completed an assessment of bias for each study included, using the Cochrane Grading of Recommendations, Assessment, Development and Evaluation (GRADE) approach. Those studies deemed to be of poor quality were not included in the final assessment.

Meta-analysis, including subgroup analysis, was pre-specified, if sufficient studies with similar endpoints had been identified.

## Results

**Figure 1:**
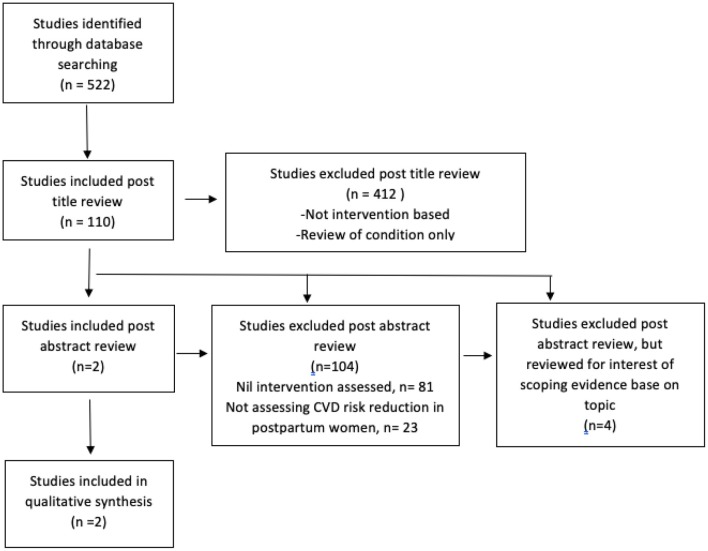
PRISMA flow chart (14).

Of 522 titles found in the initial literature search (Figure 1), only two studies were identified that met inclusion criteria (Table 2). Four other studies, whilst not meeting criteria based on study design, were also reviewed in the interests of scoping the evidence base on this topic (Table 3). A further 104 studies were reviewed however were excluded as they did not meet the inclusion criteria generally, mostly as they were not assessing a specific intervention or were not studying cardiovascular risk reduction postpartum (Table 4). These findings are summarized in the PRISMA flow diagram below (Figure 1). As described, trial registry data was also searched; this search yielded four randomized controlled trial registrations. Three are currently recruiting and one has closed recruiting and expects to report results in early 2020. These have been summarized below for the reader's information.

**Table 2:** Summary of included studies.

**Study**	**Setting**	**Study design**	**Sample size**	**Subjects**	**Trial period**	**End-points**	**Method or intervention**	**Outcomes**
The effect of calcium supplementation on blood pressure in non-pregnant women with previous pre-eclampsia: an exploratory, randomized placebo-controlled study (15)	Sub-study of WHO Calcium and Pre-eclampsia (CAP) Trial	RCT	*N* = 836 randomized *N* = 367 first visit *N* = 217 History severe PE	Non-pregnant women who had PE or eclampsia in their immediately previous pregnancy.	12 or 24 weeks after randomization	Blood pressure (systolic and diastolic)	500 mg/day Calcium or placebo	Overall trend toward decreased BP in supplemented group but NS (reduction of 1–2.5 mmHg) Statistically significant reduction in diastolic BP of severe PE group
Randomized Trial to Reduce Cardiovascular Risk in Women with Recent Preeclampsia (16)	Standalone controlled intervention trial.	RCT	*N* = 151 (assessed for eligibility *n* = 1,493)	Women with PE affected pregnancy, within 5-years of index pregnancy	9 months	Healthy diet and increase activity, change in physical in/activity, DASH diet, knowledge of risk. Weight/BP	Online intervention (educational modules, community forum, life-style coach communication) Control: Links to standard CVD risk information	High rate of access of intervention information [84% of participants accessed a minimum of 1 online module) and access to coach [89% had 3 calls with a coach) Self-reported increased knowledge of CVD risk factors (*p* = 0.01 corrected), self-efficacy for healthy eating (*p* = 0.03), less physical inactivity (*p* = 0.0006). No difference in adherence to DASH diet, sense of control of risk factors, self-efficacy for physical activity or reported physical activity. No difference in secondary outcomes (weight, blood pressure)

*RCT, Randomized Controlled Trial; WHO, World Health Organization; NS, Not significant; PE, Preeclampsia; DASH, Dietary Approaches to Stop Hypertension; CVD, Cardiovascular disease*.

**Table 3:** Summary of relevant articles not included based on study type.

	**Setting**	**Study design**	**Sample size**	**Subjects**	**Trial period**	**End-points**	**Method or intervention**	**Outcomes**
Reduction of cardiovascular risk after preeclampsia: the role of framing and perceived probability in modifying behavior (17)	Questionnaire	Survey	*N* = 175 answers *N* = 165 complete (people who declined not recorded)	Female obstetric nurses	N/A	Willingness to modify behavior (Likert scale) - Activity - Diet - Annual BP check	Survey presenting two cases (high and low risk of CVD post PE pregnancy)	Statistically significant willingness to modify behavior; affected by the perceived probability of poor outcome (*p* = 0.001).
Cardiovascular risk reduction and weight management at a hospital-based postpartum preeclampsia clinic (18)	Retrospective cohort study	Review of medical recordsSingle center (Edmonton, Alberta, CA)	*N* = 104 (initial visit) *N* = 21 completing 6 months		Minimum 6 months; Average 4.4 ± 1.4 months post-partum	BMI Physical activity	Attending dedicated, MDT, post-partum clinic (PPPEC) - Education on risks; - Assessment of risk - Weight mgmt. focus	Non-significant changes in BMI (mean weight loss 0.4 ± 4.5 kg; mean BMI decrease 0.1 ± 1.7 kg/m^2^) Significant changes in physical activity [14% prior to pregnancy, to 76% at mean 4.4 months postpartum) NB: Mean GA = 31, Mean BMI 31
Prevention of cardiovascular risk in women who had hypertension during pregnancy after 36 weeks gestation (19)	Subgroup cohort from prior RCT	Survey and risk assessment	*N* = 306 random sample from HYPITAT for risk assessment, *N* = 257 answered questionnaires	Women with hypertension affected pregnancy who participated in prior HYPITAT trial	3.5 years post-partum, 1 year after CVD risk assessment	Hypertension, BMI reduction, smoking status, lipid and BSL levels	Survey 1 year after CVD risk assessment	Reduction in self-reported smoking (42%), reduction in BMI ≥ 5% (31%)
Risk of cardiovascular disease after pre-eclampsia and the effect of lifestyle interventions: a literature -based study (20)	Literature-based study	Estimate diff in CVD risk in PE v uncomplicated pregnancy. Effects of lifestyle intervention estimated Risk prediction models used.	*N* = 16 studies included	Women with hypertension affected pregnancy and women with uncomplicated pregnancies	N/A	Primary: Cardiovascular risk after pre-eclampsia compared with an uncomplicated pregnancy Secondary: effects of lifestyle interventions on cardiovascular risk	Review of cardiovascular risk in 16 studies Calculation of difference in risk and odds ratio using risk prediction models, and calculation of risk reduction with lifestyle interventions of (exercise, dietary habits, and smoking cessation decrease)	After PE, lifestyle interventions (diet/exercise), smoking cessation, decreased CVD risk by 4–13% (OR 0.91)

*PE, Preeclampsia; CVD, Cardiovascular disease; BP, blood pressure; BMI, Body Mass Index; MDT, multidisciplinary team; BSL, blood sugar level; GA, gestational age; OR, odds ratio*.

**Table 4:** Summary of other excluded studies (*n* = 104).

**Reason for exclusion**	**Number of studies excluded**	**References**
No specific intervention assessed	81	- Preventing cardiovascular disease after hypertensive disorders of pregnancy: searching for the how and when (21). - Preeclampsia and the risk of future vascular disease and mortality: a review (22). - Counseling and management of cardiovascular risk factors after preeclampsia (23). - Long-term renal and cardiovascular risk after preeclampsia: toward screening and prevention (24) - Health workers' knowledge on future vascular disease risk in women with pre-eclampsia in south western Nigeria (25). - Determinants of future cardiovascular health in women with a history of preeclampsia (26). - Pregnancy characteristics and women's future cardiovascular health: an underused opportunity to improve women's health? (27) - Risk of future cardiovascular disease in women with prior preeclampsia: a focus group study (28). - Cardiovascular disease in menopause: does the obstetric history have any bearing? (29) - Postpartum evaluation and long-term implications (30). - 10-Year cardiovascular event risks for women who experienced hypertensive disorders in late pregnancy: the HyRAS study (31). - Preeclampsia and future cardiovascular disease in women: how good are the data and how can we manage our patients? (32) - Hypertension in pregnancy and later cardiovascular risk: common antecedents? (33) - Hypertensive disorders in pregnancy and subsequently measured cardiovascular risk factors (34). - Preeclampsia and cardiovascular risk: general characteristics, counseling and follow-up (35). - How should women with pre-eclampsia be followed up? New insights from mechanistic studies (36). - Pregnancy: a screening test for later life cardiovascular disease (37). - The unchartered frontier: preventive cardiology between the ages of 15 and 35 years (38). - Women-specific factors to consider in risk, diagnosis and treatment of cardiovascular disease (39). - The Maternal Health Clinic: a new window of opportunity for early heart disease risk screening and intervention for women with pregnancy complications (40). - Pre-eclamptic pregnancies: an opportunity to identify women at risk for future cardiovascular disease (41). - Similarities between pre-eclampsia and atherosclerosis: a protective effect of physical exercise? (42) - Preeclampsia: a challenge also for Cardiologists (43). - Blood pressure profile 1 year after severe preeclampsia (44). - Preventing stroke and assessing risk in women (45). - Preeclampsia: pathogenesis, prevention, and long-term complications (46). - Hypertension in pregnancy and future maternal health (47). - Association of pre-eclampsia with metabolic syndrome and increased risk of cardiovascular disease in women: a systemic review (48). - Risk factors of hypertensive disorders among Chinese pregnant women (49). - Alterations to the maternal circulating proteome after preeclampsia (50). - Cardiovascular implications of preeclampsia (51). - Pregnancy: window into women's future cardiovascular health (52). - Adverse pregnancy outcomes and cardiovascular risk factor management (53). - Pregnancy as a window to future health (54). - Preeclampsia: short-term and Long-term Implications (55). - Present status of clinical care for postpartum patients with hypertensive disorders of pregnancy in Japan: findings from a nationwide questionnaire survey (56). - Cardiovascular risk factors 1 year after a hypertensive disorder of pregnancy (57). - Risk factors of hypertensive pregnancies in women with diabetes and the influence on their future life (58). - Hypertension in pregnancy greater risk than previously thought (59). - Is there a relationship between pregnancy induced hypertension and obstructive sleep apnea? Case report (60). - Cardiovascular risk, lipids and pregnancy: preeclampsia and the risk of later life cardiovascular disease (61). - Renovascular prognosis of preeclampsia on the mother and the child (62). - Preeclampsia as cardiovascular risk factor (63). - Importance of engaging obstetrician/gynecologists in cardiovascular disease prevention (64). - Hypertensive pregnancy disorders as a risk factor for future cardiovascular and metabolic disorders (65). - Biochemical cardiovascular risk factors after hypertensive pregnancy disorders: a systematic review and meta-analysis (66). - Cardiovascular risk management after a hypertensive disorder of pregnancy (67). - Ten-year, thirty-year, and lifetime cardiovascular disease risk estimates following a pregnancy complicated by preeclampsia (68). - Preeclampsia (marker of chronic kidney disease): from genesis to future risks (69). - Prevention of vascular dysfunction after preeclampsia: a potential long-term outcome measure and an emerging goal for treatment (70). - Gestational hypertension: a neglected cardiovascular disease risk marker (71). - Preeclampsia as a female-specific risk factor for chronic hypertension (72).
		- Role of pre-eclamptic toxemia or eclampsia in hypertensive women attending cardiac clinic of Ahmadu Bello University Teaching Hospital Zaria, Nigeria (73). - Pregnancy-related hypertension: a cardiovascular risk situation (74). - Investigating the risk of hypertension shortly after pregnancies complicated by preeclampsia (75). - Cardiovascular sequelae of preeclampsia/eclampsia: a systematic review and meta-analyses (5). - Recognizing pregnancy-associated cardiovascular risk factors (76). - Ethnic and racial disparities in hypertension management among women (77). - Cardiovascular disease screening (78). - Heart, arteries and women, a care pathway for women at high cardiovascular risk (79). - Cardiovascular risk in women: focus on hypertension (80). - Pregnancy as a window to future health (81). - Aspirin use in women: current perspectives and future directions (82). - Maternal deaths due to hypertensive disorders in pregnancy (83). - Pregnancy risks associated with obesity (84). - Clinical applications of biomarkers in preeclampsia (85). - Nitrergic system and plasmatic methylarginines: evidence of their role in the perinatal programming of cardiovascular diseases (86). - Cardiovascular disease in women: primary and secondary cardiovascular disease prevention (87). - Effects of preeclampsia on maternal and pediatric health at 11 years postpartum (88). - Early gestational age at preeclampsia onset is associated with subclinical atherosclerosis 12 years after delivery (89). - Effect of early-onset preeclampsia on cardiovascular risk in the fifth decade of life (90). - Hypertension in pregnancy is associated with elevated homocysteine levels later in life (91). - Preeclampsia and cardiovascular disease death: prospective evidence from the child health and development studies cohort (92). - Cardiology for gynecologists–a mini review (93). - Stroke in women–oral contraception, pregnancy, and hormone replacement therapy (94). - Hypertension in pregnancy and women of childbearing age (95). - Chronic hypertension in pregnancy: diagnosis, management, and outcomes (96). - To prevent cardiovascular disease, pay attention to pregnancy complications (97).
Not studying cardiovascular risk reduction in women post-partum	23	- The potential role of statins in preeclampsia and dyslipidemia during gestation: a narrative review (98). - Preventing deaths due to the hypertensive disorders of pregnancy (99). - Prevention of perinatal death and adverse perinatal outcome using low-dose aspirin: a meta-analysis (100). - Guided imagery for treating hypertension in pregnancy (101). - Interventions for treating pre-eclampsia and its consequences: generic protocol (102). - Safety and pharmacokinetics of pravastatin used for the prevention of preeclampsia in high-risk pregnant women: a pilot randomized controlled trial (103). - Blood pressure patterns and body mass index status in pregnancy: an assessment among women reporting for antenatal care at the Korle-Bu Teaching hospital, Ghana (104). - Systematic Review of Vitamin D and Hypertensive Disorders of Pregnancy (105). - Potent Vasoconstrictor Kisspeptin-10 Induces Atherosclerotic plaque progression and instability: reversal by its receptor GPR54 antagonist (106). - Exercise during pregnancy and risk of gestational hypertensive disorders: a systematic review and meta-analysis (107). - The performance of risk prediction models for pre-eclampsia using routinely collected maternal characteristics and comparison with models that include specialized tests and with clinical guideline decision rules: a systematic review (108). - Selenium status in U.K. pregnant women and its relationship with hypertensive conditions of pregnancy (109). - Pre-eclampsia, eclampsia, and hypertension (110). - Hypertension in pregnancy (111). - Pregnancy Outcomes Associated with Stage 1 Hypertension in a High-Risk Cohort (112). - Antiplatelet agents and anticoagulants for hypertension (113). - First-line drugs for hypertension (114). - Calcium supplementation for prevention of primary hypertension (115). - Pharmacotherapy for hypertension in adults aged 18 to 59 years (116). - Reducing women's cardiovascular disease risk profile (117). - How to stay heart healthy in 2011: considerations for the primary prevention of cardiovascular disease in women (118). - Comprehensive primary prevention of cardiovascular disease in women (119). - Identifying and managing younger women at high risk of cardiovascular disease (120).
Non-randomized study (i.e., not appropriate study type]	6	- To prevent cardiovascular disease, pay attention to pregnancy complications (97). - Identifying and managing younger women at high risk of cardiovascular disease (120). - Comprehensive primary prevention of cardiovascular disease in women (119). - Cardiology for gynecologists–a mini review (93).
		- Stroke in women–oral contraception, pregnancy, and hormone replacement therapy (94). - Pregnancy outcomes associated with stage 1 hypertension in a high-risk cohort (112, 121).
Included women with other primary cause for hypertension or essential hypertension	9	- Cardiovascular disease in women: primary and secondary cardiovascular disease prevention (87). - Reducing women's cardiovascular disease risk profile (117). - How to stay heart healthy in 2011: considerations for the primary prevention of cardiovascular disease in women (118). - Hypertension in pregnancy and women of childbearing age (95). - Chronic hypertension in pregnancy: diagnosis, management, and outcomes (96). - First-line drugs for hypertension (114). - Pharmacotherapy for hypertension in adults aged 18 to 59 years (116). - Antiplatelet agents and anticoagulants for hypertension (113). - Calcium supplementation for prevention of primary hypertension (115).
Pregnancy not affected by PE or GH only	1	- Hypertensive pregnancy in diabetes–risk factors and influence on future life (58).
>10 years postpartum	5	- Effects of preeclampsia on maternal and pediatric health at 11 years postpartum (88). - Early gestational age at preeclampsia onset is associated with subclinical atherosclerosis 12 years after delivery (89). - Effect of early-onset preeclampsia on cardiovascular risk in the fifth decade of life (90). - Hypertension in pregnancy is associated with elevated homocysteine levels later in life (91). - Preeclampsia and cardiovascular disease death: prospective evidence from the child health and development studies cohort (92).

*Studies may have more than one reason for exclusion. The table above shows the primary reason for exclusion, assessed in a sequential fashion, as follow: (1) No intervention assessed; (2) Not assessing CVD risk reduction in postpartum women; (3) Non-randomized study (i.e., not appropriate study type); (4) Included women with other primary cause for hypertension and essential hypertension; (5) Pregnancy not affected by PE or GH only; (6) >10 years postpartum*.

As only two randomized controlled trials were identified, with largely heterogeneous outcomes, a qualitative (narrative) synthesis only was performed.

RCT-1 was a sub-group analysis of the larger WHO Calcium and preeclampsia study (CAP) (15) which in itself is a subset of the PRE-EMPT (Pre-eclampsia, Eclampsia, Monitoring, prevention, and Treatment) study. This trial was a placebo-controlled trial investigating the pharmacological intervention of the addition of 500 mg/day of calcium to non-pregnant women who have a low dietary calcium intake. Eight hundred and thirty-six women were randomized however a complete dataset was only available for 201 women.

This was a multicenter trial based in South Africa, Zimbabwe, and Argentina. Inclusion criteria were limited to non-pregnant women whose most recent pregnancy had been affected by preeclampsia. Calcium was commenced outside of pregnancy at the time of randomization and continued until 20 weeks' gestation if another pregnancy occurred. The placebo group received an identical tablet. At 20 weeks' gestation, all women were switched to unblinded calcium in accordance with WHO guidelines. Follow-up was at recruitment and then every 12 weeks until the next pregnancy. Recruitment fell short of the power calculation however baseline characteristics were the same across both groups.

A non-statistically significant trend toward a decrease in blood pressure at follow-up was found (reduction of 1–2.5 mgHg). There was a statistically significant reduction in diastolic blood pressure of the sub-group of women who had a pregnancy previously affected by severe preeclampsia (defined here as eclampsia, HELLP syndrome, systolic blood pressure >160 mmHg, diastolic blood pressure >110 mmHg, onset earlier than 28 weeks gestation or ICU admission). In this group, there was a mean difference in diastolic blood pressure between the treatment group and placebo of −3.4 mmHg (95% CI −0.4–6.4, *p* = 0.025). Compliance was noted to be an average of 80% across all groups.

RCT-2 was identified at final search immediately prior to finalization of this analysis. Heart Health 4 Moms (HH4M) (16) tested an online intervention to modify lifestyle risk factors in women who had recently been diagnosed with preeclampsia with a view to reducing cardiovascular risk.

Participants were recruited from the United States of America via online advertising on social media in both English and Spanish. Participants were included if the index PE affected pregnancy was within the preceding 5 years, if they had a normal current blood pressure and BMI between 18.5 and 40 kg/m^2^, if they had internet access and could communicate in English or Spanish at an eighth-grade level. Any women with active or past medical problems (including but not limited to diabetes, kidney disease or cardiovascular disease), who was currently pregnant, who had a history of bariatric surgery or who was taking any anti-hypertensive medications or medications affecting weight were excluded. The control group was given access to the Control-HH4M website which contained standard, publicly available information on reduction of CVD risk. The intervention group had access to the intervention website which included modules regarding risk reduction specific to their condition, details on a specifically recommended diet, and physical activity; on completion of these modules the participant was acknowledged with an online “badge.” Direct access to a life-style coach was also arranged that was provided by way of scheduled phone-calls and personalized emails. Three assessments were carried out through the 9 months programme. Validated questionnaires were used. Data was missing for 0–9% of each primary outcome and 19% for the Dietary Approaches to Stop Hypertension (DASH)-frequency questionnaire.

After screening of 1,493 women, 151 were eventually included and randomized (76 to intervention and 75 control). At baseline, approximately half of women in each group were <12 months since the affected pregnancy, and for the majority it had been their first baby. Baseline demographics suggest that a higher socio-demographic group than the USA average was recruited, as over 90% of both groups had at least some post-high school education, and only 3% were African American. During the intervention, 69% of controls accessed the control website and 99% accessed the intervention website with good retention rates at the completion of the study (93 and 91%, respectively). Results showed the participants' self-efficacy in eating a healthy diet improved significantly (*p* = 0.03) in the intervention group; as did a decrease in physical inactivity (e.g., watching television) (*p* = 0.0006). Intervention participants also felt better informed regarding their risk profile (*p* = 0.01). However, there were no significant differences in participation in physical activity, adherence to the recommended diet and secondary outcomes of self-reported weight and blood pressure remained unchanged.

### Characteristics of Excluded but Relevant Studies (Table 3)

Four studies identified comprised largely observational studies. Janmohamed et al. (18) was a retrospective cohort study, assessing outcomes from a dedicated multidisciplinary preeclampsia follow-up clinic at the study hospital designed to focus on lifestyle modifications known to reduce the risk of hypertension. Twenty-one of 104 women completed 6 months of follow-up. The women in this study mostly represented a high-risk cohort with early-onset preeclampsia, in that average gestation at birth of the index pregnancy was 31 weeks and women had a mean starting BMI of 31. There was a non-statistically significant trend toward a decrease in BMI and increase in physical activity.

van Kesteren et al. (19) examined the effect of cardiovascular risk assessment 2.5 years postpartum on health behaviors 12 months later (i.e., at 3.5 years postpartum) in a subgroup of women who participated in the HYPITAT I trial of timing of delivery in women with gestational hypertension or preeclampsia at 36 weeks gestation or more (122). A random sample of 306 women from HYPITAT were invited to attend a cardiovascular risk assessment 2.5 years postpartum [HyRAS study (31)], with results fed-back to the women by way of a letter detailing their risk. One year later, women were invited to complete a follow-up questionnaire regarding their behaviors, which 257 of the 306 completed. This study showed some lifestyle behavior improvements over time, particularly a decrease in BMI >5 in 31% of previously obese women and 42% of women who reported smoking had quit. Of women who had established hypertension at 2.5 years (91 women), 36% were taking antihypertensive agents at the time of questionnaire follow-up. This study suggests that increasing awareness of risk can promote modest change without any specific lifestyle behavior change or pharmacological intervention.

Bokslag et al. (17) was a prospective questionnaire-based study which focused on women's intentions regarding modification of behavior when presented with two clinical cases suggestive of low or high-risk chance of developing a long-term increase in cardiovascular risk. Study participants were limited to female obstetric nurses of childbearing age. The Likert scale was used to assess willingness to modify behavior (activity, diet, annual BP checks). The study showed a statistically significant willingness toward modifying behavior when considering the high-risk case suggestive of a higher chance of a poor outcome.

We also identified one relevant meta-analysis, published in 2013 (20). This analysis aimed to estimate the difference in the risk of long-term CVD in preeclampsia against an uncomplicated pregnancy and the effect of lifestyle interventions, through extrapolation from lifestyle studies performed in other settings and populations (including five studies that had between 35 and 69% female participation). Sixteen studies were included for the post PE analysis. The range in time after follow-up was 1.0–19.0 years (majority of studies <10 years). Appropriate risk prediction models were employed to determine life-time risk of CVD events. After adjustments, it was concluded that lifestyle interventions (diet/exercise) and smoking cessation would be likely to decrease the overall cardiovascular risk by 4–13% in women who had a pregnancy complicated by PE.

### Relevant but Not Yet Reported Studies

The following four trials would meet the criteria for this analysis however are not yet completed. A summary of the methodology of each is provided herein. Together, these studies are expected to yield a further 660 individual patient datasets for analysis.

“RedCarRisk” (123) is a study aimed at detecting an objective improvement in arterial stiffness after engagement with a 6 months conditional workout programme with midwifery support after a PE affected pregnancy. This study has closed recruiting and is due to report outcomes in 2020.

“Heart Health 4 New Moms (HH4NM)” (112) is similar in design to the original HH4M trial described above. This pilot trial will focus on recruitment of overweight and obese women in the first year after their PE-affected pregnancy. It will randomize to an online lifestyle-based intervention to improve weight-loss at 1 year postpartum and will include home blood pressure monitoring. The trial will investigate the feasibility of the intervention as well as the effect of home blood pressure monitoring on progression to chronic hypertension. This study is currently recruiting.

“BP2” (124) is a 3-arm post-partum intervention trial targeting the first 12 months after a pregnancy affected by a hypertensive disorder. The arms comprise: (1) optimized usual care, (2) brief education (30 min consultation with physician and then dietician), and (3) extended lifestyle intervention (as for group 2 with enrolment in the NSW Get Healthy Service for intensive lifestyle behavior change coaching over 6 months). Primary outcomes include change in blood pressure, weight and waist circumference (clinically recorded) and secondary outcomes include self-reported changes (validated questionnaire), serological measures, vascular function, maternal satisfaction with the programme and cost-effectiveness. This trial is currently recruiting.

“Be Healthy for your Heart (BH4YH)” (125) is a postpartum intervention trial where participants will be randomized to either access to the National Heart Foundation website (control group) or specific online resources developed by the trial team that will require participant interaction in goal setting and monitoring progress with lifestyle modifications over a 3 months course. Primary end-points include acceptability and resource efficiency. Secondary endpoints include objective measures of blood pressure, diet, and routine serology as well as life-quality questionnaires. This trial is currently recruiting.

## Discussion

Current guidelines acknowledge the long-term CVD risk associated with hypertensive disorders of pregnancy and currently recommend long-term follow-up for cardiovascular risk factor assessment and management, as well as adoption of a healthy lifestyle (3, 126). However, guidance on specific postpartum interventions to decrease cardiovascular risk is lacking. This systematic review identified a paucity of RCTs on postpartum interventions to reduce CVD risk after PE or GH. What evidence was available from the two included studies, and the relevant but excluded studies, suggests that implementing CVD risk factor and lifestyle education and intervention strategies, either online or face to face, has a modest but appreciable effect in changing CVD risk factor incidence in affected women. However, this requires confirmation from the several registered but not yet reported RCTs in this space.

Pregnancy has long considered to be a stress-test unique to the female population. The development of particular diseases during this confined period where the body is expending energy at more than twice its non-pregnant metabolic rate (127) and sees marked changes in maternal physiology offers a window into the future health risks particular to that woman. As a result, interventions to reduce the cardiovascular risk posed by PE and GH are required.

The Hofmeyr et al. study is suggestive that the pharmacological intervention of calcium supplementation in those deficient in dietary calcium may be beneficial in lowering blood pressure long term. However, this study was underpowered for the primary outcome and unfortunately suffered a high attrition rate. Moreover, the reductions were modest and their ability to be borne out over a period longer than 12 weeks has not been proven. The HH4M trial suggests that online education and lifestyle interventions show promise in promoting healthy behaviors after preeclampsia. However, although not suffering the high attrition rate of the Hofmeyr et al. study, HH4M had a high number of women screened for each inclusion (~10–1). It is also of some concern that included women overall represented a highly-educated group with under-representation of African Americans, the group with the worst maternal health outcomes in the USA (128). It would be important to ensure that in a wider implementation of online interventions, efforts are made to be as inclusive as possible of all at-risk women.

The failure to identify further completed trials may be as a result of the emerging nature of this research. A trial to identify a change in actual cardiovascular disease outcomes would be required to have a large enough power to show a significant reduction in risk in an outcome that is not expected for many years following the index pregnancy. In addition, the follow-up period would need to be up to several decades long in order to capture the population at the time of the index cardiac event, rendering such a trial a challenge to conduct.

Shorter follow-up studies focusing on hypertension, and other risk factors and surrogate markers as end-points for CVD, are therefore more realistic. Both some of the non-randomized research found and the registered but not yet completed RCTs are focusing on these populations. Interventions including the development of a close monitoring system and linkage with a regular healthcare provider, lifestyle modifications (diet and exercise to aid in weight loss and general health) whether internet-based or phone based in conjunction with follow-up of neonatal and infant development are all currently under study. These can all potentially act as the starting point for a longer follow-up study. These ongoing studies are expected to yield a further 660 cases from which to draw experience.

As noted in the RCTs reviewed, enrolling an appropriately powered cohort can be challenging. Reasons for this are likely associated with the nature of the disease: as time away from the index pregnancy increases, the perceived risk and need to continue intervention can decrease as women do not necessarily “feel” particularly unwell and the experience of complicated, high risk pregnancy becomes far removed. Challenges noted in the Janmohamed 2015 study, particularly maintaining participation in follow-up are of particular interest in this case: if women are not educated regarding the ongoing risk to themselves in the long-term, the impetus to maintain follow-up with risk reduction practices is not felt. Discussed studies show that when provided with structured education on risk, the information is retained. This may be a simple first step at the time of delivery that could help to involve women in long-term follow-up studies aimed at risk reduction interventions.

Education of health care providers has been demonstrated to be effective in the Bokslag 2016 study. Whilst the findings of “willingness to change” in the cohort surveyed was clear, the cohort was somewhat biased given a presumed baseline level of knowledge regarding physiology and natural history of disease as a result of occupation (obstetric nurses). This does show, however, if an appropriate level of education can be attained in the target population, perhaps a similar “willingness” may be elicited and in turn, a change in behavior affected. Indeed, a working party in conjunction with the Heart Foundation (Australia) have developed a patient information sheet outlining the long term risks of having had an HDP affected pregnancy and basic steps to start to mitigate that risk (129). While awaiting high-level evidence to support appropriate postpartum interventions to decrease women's risk of CVD after GH/PE, this approach to information provision, risk factor assessment, and encouragement of healthy lifestyle, appears unlikely to do harm and a reasonable strategy to pursue.

## Conclusion

There is a paucity of intervention trials conducted attempting to decrease the increased cardiovascular risk of women who have been affected by a hypertensive disorder of pregnancy. Current studied interventions focusing on lifestyle modification by way of prescribed (online) programmes have had some effect in reduction of secondary end-points for cardiovascular disease risk. This corroborates current guidelines in the management of these long-term risks. Given the significant burden to women's health presented by long term CVD, appropriately powered randomized trials of further interventions to reduce this risk in this particular group are an important area for future research.

## Author Contributions

AH and NL contributed to the conception and design of the study. NL and GJ carried out the search strategy independently. NL and AH contributed to the analysis of the included studies. NL wrote the draft manuscript. All authors contributed to manuscript revision, read, and approved the submitted version.

### Conflict of Interest

The authors declare that the research was conducted in the absence of any commercial or financial relationships that could be construed as a potential conflict of interest.

## Appendix 1: Search Terms

Search strategy:

1 hypertension, pregnancy-induced OR pre-eclampsia OR gestational hypertension OR “pregnancy AND hypertension”

2 risk factors OR causality

3 cardiovascular diseases OR pregnancy complications, cardiovascular OR cardiovascular diseases

[prevention and control]

4 risk reduction intervention.mp. or *Risk Reduction Behavior

5 limit to year “2008–Current.”
